# P-282. Cost Effectiveness Analysis of Ceftolozane/Tazobactam for the Treatment of Patients with Ventilated Hospital-Acquired Bacterial Pneumonia and Ventilator-Associated Bacterial Pneumonia in the Philippines

**DOI:** 10.1093/ofid/ofae631.485

**Published:** 2025-01-29

**Authors:** Geovin Dexter c Uy, Karlo Paredes, Gillian Camille Abello, Carolyn Cameron, Joe Yang

**Affiliations:** MSD Philippines, Makati City, National Capital Region, Philippines; MSD Philippines, Makati City, National Capital Region, Philippines; MSD Philippines, Makati City, National Capital Region, Philippines; MSD Australia, Sydney, New South Wales, Australia; Merck Co., Upper Gwynedd, Pennsylvania

## Abstract

**Background:**

Ceftolozane/tazobactam (C/T) was licensed by Philippine Food and Drug Administration for the treatment of ventilated hospital-acquired bacterial pneumonia (vHABP) and ventilator-associated bacterial pneumonia (VABP) last 2019. Increasing resistance rates to antibiotics available in the Philippine National Formulary (PNF) by Pseudomonas aeruginosa associated vHABP/VABP has concerned clinicians due to its overall morbidity and mortality. This study assessed the cost-effectiveness of C/T treatment compared with existing antimicrobials among adult patients with confirmed susceptibility profile of Pseudomonas aeruginosa associated vHABP/VABP.

**Methods:**

An economic model was developed to compare the lifetime costs and quality-adjusted life-years (QALYs) associated with C/T and the currently listed antimicrobials (meropenem, aminoglycosides, colistin) in the PNF for the treatment of patients with Pseudomonas aeruginosa associated vHABP/VABP. A short-term decision tree was used for the in-hospital period, followed by a long-term Markov model to depict lifetime costs and outcomes.

Pathogen susceptibility was sourced from the Program to Assess Ceftolozane/Tazobactam Susceptibility (PACTS) database; clinical efficacy was obtained from the published literature. The model assumed a public payer perspective, capturing direct costs incurred by the health system, and direct health outcomes for treated patients. Discount rates applied followed local HTA methods guide.

**Results:**

On treating Pseudomonas aeruginosa associated vHABP/VABP, C/T was associated with a higher absolute cure rate (17%) and lower absolute mortality rate (-3%) than meropenem. The incremental cost-effectiveness ratio (ICER) for C/T compared to meropenem, amikacin, and colistin were ₱267,396, ₱164,570, and ₱47,504 per QALY gained, respectively. C/T generated more QALYs than exiting antimicrobials at an increased cost per patient, but resulting in ICER less than one times the Gross Domestic Product (GDP) of ₱233,902 for amikacin, and colistin, and ∼ 1.2x GDP per capita for meropenem.

**Conclusion:**

C/T represents a cost-effective treatment for payers and an important option to be listed in the PNF for clinicians treating patients with Pseudomonas aeruginosa vHABP/VABP in the Philippines.

**Disclosures:**

**GEOVIN DEXTER C. UY, RPh, MPH**, MSD Philippines: employee|MSD Philippines: Stocks/Bonds (Private Company) **Karlo Paredes, RN, MPH, PhD (cand)**, MSD Philippines: employee|MSD Philippines: Stocks/Bonds (Private Company) **Gillian Camille Abello, MD**, MSD Philippines: employee **Carolyn Cameron, MPH**, MSD Australia: employee|MSD Australia: Stocks/Bonds (Private Company) **Joe Yang, PhD**, Merck & Co., Inc: employee|Merck & Co., Inc: Stocks/Bonds (Private Company)

